# TM4SF1 promotes esophageal squamous cell carcinoma metastasis by interacting with integrin α6

**DOI:** 10.1038/s41419-022-05067-2

**Published:** 2022-07-14

**Authors:** Sicong Hou, Xin Hao, Jiajia Li, Siwei Weng, Jiaxin Wang, Tiantian Zhao, Wenqian Li, Xiaolin Hu, Bing Deng, Jianguo Gu, Qinglei Hang

**Affiliations:** 1 Department of Gastroenterology, The Affiliated Hospital of Yangzhou University, Yangzhou University, 225009 Yangzhou, Jiangsu China; 2 Department of Clinical Medicine, Medical College, Yangzhou University, 225001 Yangzhou, Jiangsu China; 3 Department of stomatology, Clinical Traditional Chinese Medicine College of Yangzhou University, 225000 Yangzhou, Jiangsu China; 4 Division of Regulatory Glycobiology, Institute of Molecular Biomembrane and Glycobiology, Tohoku Medical and Pharmaceutical University, Sendai, Miyagi 981-8558 Japan; 5 Department of Experimental Radiation Oncology, The University of Texas MD Anderson Cancer Center, Houston, TX 77030 USA

**Keywords:** Oesophageal cancer, Integrins

## Abstract

Transmembrane-4 L-six family member-1 (TM4SF1) is a member of the L6 family and functions as a signal transducer to regulate tumor cell behaviors. However, the function and mechanism of TM4SF1 in esophageal squamous cell carcinoma (ESCC) metastasis remains unclear. Here, we find that TM4SF1 expression is increased and positively correlated with clinical TNM stage, N classification, differentiation, tumor size, and poor prognosis in ESCC patients. Interestingly, we demonstrate that TM4SF1 promotes ESCC cell adhesion, spreading, migration, and invasion, but not cell proliferation, in a laminin-dependent manner by interacting with integrin α6. Mechanistically, the TM4SF1/integrin α6/FAK axis signal pathway mediates cell migration under laminin-coating condition. Inhibiting FAK or knocking down TM4SF1 can attenuate TM4SF1-mediated cell migration and lung metastasis. Clinically, the TM4SF1/integrin α6/FAK axis positively correlates with ESCC. Altogether, these findings reveal a new mechanism of TM4SF1 in promoting ESCC metastasis via binding to integrin α6 and suggest that the cross-talk between TM4SF1 and integrin α6 may serve as a therapeutic target for ESCC.

## Introduction

Esophageal cancer (EC) is the 7th most commonly diagnosed solid malignant tumor worldwide and the 6th leading cause of cancer-related deaths, approximately 90% of which are esophageal squamous cell carcinoma (ESCC) in China [1]. Although significant improvements have been made in diagnostic techniques and comprehensive therapy, the overall 5-year survival rate of the disease remains poor at approximately 20% on recurrence, extensive invasion, and metastasis [2].

Cancer metastasis is a complicated multi-step process that requires numerous specific molecular interactions contributed by the tumor cell transmembrane proteins, the surrounding extracellular matrix (ECM), and stromal cells. The tetraspanin superfamily is a group of transmembrane proteins located in the plasma membrane and has received much attention for its role in cancer cell motility and the cell-microenvironment interaction [3, 4]. Transmembrane-4-L-six-family member-1 (TM4SF1) is a member of the tetraspanin superfamily with four transmembrane domains and functions as a signal transducer involved in various cellular activities [5–7]. TM4SF1 may interact with tetraspanins, integrins, immunoproteins, and PDZ-domain-containing proteins to form tetraspanin-enriched microdomains (TEMs), which play vital roles in modulating cellular functions, such as cell adhesion, migration, invasion through various signaling pathways [8–10]. For instance, the unconventional PDZ-domain-binding motif (X-Tyr-X-Cys) in the C-terminal cytoplasmic portion of TM4SF1 can associate with syntenin-2 and further activates syntenin-1 to participate in cancer cell proliferation and migration [8]. In addition, a recent report shows that collagen I could enhance TM4SF1-discoidin domain receptor 1 (DDR1) interaction by inducing DDR1 aggregation and recruitment of TM4SF1, thus forming TEMs to activate intracellular signaling pathways, which implies that matrix protein may be crucial for TEMs formation and thereby promote tumor metastasis [11]. Although accumulating evidence suggests that overexpressed TM4SF1 can contribute to the development, progression, and drug resistance of many malignant tumors, the biological functions and involved mechanisms of TM4SF1 in ESCC remain largely unknown. So far, one study demonstrates that miR-141-mediated upregulation of TM4SF1 could stimulate self-renewal of esophageal cancer stem-like cells, suggesting the potential involvement of TM4SF1 in EC malignancy [12]. Therefore, a better understanding of biological functions and mechanisms of TM4SF1 in ESCC is essential for developing a biomarker or therapeutic target.

Integrins are a family of heterodimeric transmembrane proteins consisting of α and β subunits that plays essential roles in carcinogenic process and malignant progression [13]. In humans, 18 α and 8 β subunits form 24 distinct heterodimers to facilitate cell-to-ECM adhesive interactions by diverse ECM ligand combinations, such as collagen, fibronectin, laminin, and vitronectin [14]. Alterations in the expression of specific integrins, such as integrin α5, α6, β1, β4, and β6, have been observed in EC and correlates with aggressive features and poor prognosis [15–17]. At a molecular level, the sophisticated networks of integrins and other membrane receptors contribute to its activation, internal signaling cascades, and thereby play a critical role in cancer progression and metastasis [18]. For example, cross-talk between integrin α6β4 and epidermal growth factor receptor (EGFR) promotes breast cancer progression by inducing EGFR clustering and Rho activation [19]. However, the detailed mechanism of integrins regulating malignancy in ESCC is poorly understood.

In the present study, we uncover a novel mechanism of TM4SF1 in promoting ESCC cell migration and metastasis via cross-talking with integrin α6. Tissue microarrays (TMA) show that TM4SF1 expression is strongly higher in ESCC tissues than in non-tumor tissues, and upregulated TM4SF1 is significantly associated with TNM stage, N classification, differentiation, tumor size, and poor overall survival. TM4SF1 knockdown dramatically suppresses cell adhesion, spreading, and migration, but not proliferation, in a laminin-dependent manner. In a mechanistic manner, we demonstrate that TM4SF1 can interact with integrin α6 to promote ESCC cell migration via activation of the FAK/PI3K/AKT pathway on laminin-coating condition. Furthermore, we identify that TM4SF1-integrin α6 complex is essential for activating FAK signaling and its-mediated cell migration on laminin. Moreover, pharmacological inhibition of FAK signaling with VS-4718 efficiently blocks TM4SF1-integrin α6 induced cell migration and cellular signaling. Consistent with the in vitro study results, the TM4SF1/integrin α6/FAK signaling axis is required for ESCC cell metastasis through lung colonization model in vivo. Our data reveal a pivotal role of TM4SF1-integrin α6 complex in modulating laminin-dependent ESCC cell migration and metastasis via FAK/PI3K/AKT signaling pathway, highlighting that TM4SF1 has potential as a prognostic factor and therapeutic target for ESCC.

## Results

### TM4SF1 is upregulated in ESCC tissues and correlated with poor prognosis

**Fig. 1:**
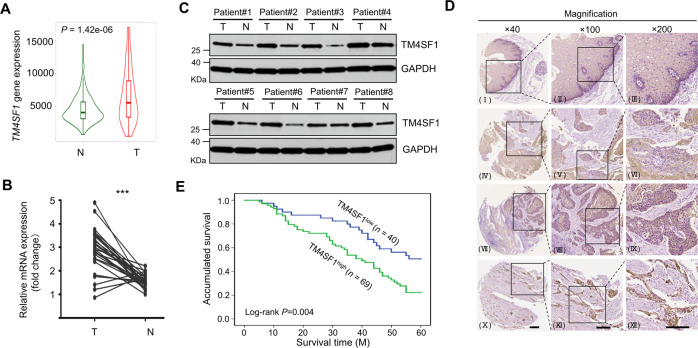
High TM4SF1 expression is significantly associated with poor prognosis. **A** The violinplot of TM4SF1 gene expression in EC tissue (T, *n* = 418) when compared with the normal esophageal tissue (N, *n* = 161), the data was generated via TNMplot. **B** The mRNA levels of *TM4SF1* in ESCC tissues and paired esophageal tissues were determined by qPCR. Graphic representation of the fold increases of mRNA in ESCC tissues (T) compared to paired esophageal tissues (N). The quantitative data were statistically analyzed as means ± s.d. (*n* = 27; ***P < 0.001, by paired *t* test). **C** Lysates from 8 paired ESCC samples (T) and adjacent normal tissues (N) were immunoblotted by anti-TM4SF1 antibody, GAPDH was used as a loading control. **D** Representative photos of TM4SF1 expression in ESCC tissues and non-cancerous tissues using TMA sections. IHC stainings of TM4SF1 in the non-cancerous specimen (I, II, and III), highly differentiated ESCC specimen (IV, V, and VI), moderately differentiated ESCC specimen (VII, VIII, and IX), and poorly differentiated ESCC specimen (X, XI, and XII). Original magnifications are ×40 in I, IV, VII, and X. Original magnifications are ×100 in II, V, VIII, and XI. Original magnifications are ×200 in III, VI, IX, and XII. Scale bar, 200 μm. **E** Kaplan–Meier analysis of overall survival in a cohort of ESCC patients (*n* = 109). The cumulative survival rate in patients with TM4SF1 highly-expressed (green line) is significantly lower than that in lowly-expressed ones (blue line). Statistical significance was determined by a log-rank test, *P* = 0.004.

To investigate the role of TM4SF1 in ESCC, the mRNA expression level of *TM4SF1* in tumor and normal tissues was compared in the TNMplot online database (https://www.tnmplot.com/) as described before [20]. Interestingly, the mRNA expression level of *TM4SF1* was significantly upregulated in EC compared with the normal tissues (Fig. 1A). We performed the qPCR and WB analyses by using paired ESCC and adjacent normal tissues to confirm this. Consistently, the upregulated expression of TM4SF1 was also observed in the ESCC tissues on both the mRNA (Fig. 1B, 27 paired) and the protein levels (Fig. 1C, 8 paired). These results indicate that TM4SF1 may be serve as an oncogene in ESCC.

In addition, the relationship between TM4SF1 expression and clinicopathologic characteristics in 109 ESCC patients was also analyzed according to the IHC results. As shown in Supplementary Table 1, TM4SF1 expression was positively correlated with TNM stage (*P* = 0.016), N classification (*P* = 0.027), differentiation (*P* = 0.031) and tumor size (*P* = 0.023), but not with gender, age, smoking history, and T classification (*P* > 0.05). Consistently, poorly differentiated ESCC specimens exhibited strong staining of TM4SF1 (Fig. 1D, X-XII). By contrast, moderately and highly differentiated ESCC specimens only exhibited moderate and low staining, respectively (Fig. 1D, IV-IX). Moreover, the correlation between TM4SF1 and patient survival was analyzed in 109 patients, and the Kaplan–Meier survival curve of low versus high expression of TM4SF1 showed a highly significant separation (Fig. 1E, *P* = 0.004). Furthermore, Multivariate analysis using the Cox proportional hazards model showed that TM4SF1 (*P* = 0.006) and TNM stage (*P* = 0.003) were independent prognostic indicators of overall survival (Supplementary Table 2). These results indicate that aberrant expression of TM4SF1 may account for the aggressive nature of ESCC and poor prognosis.

### TM4SF1 promotes invasion, but not migration and proliferation, in ESCC cells under normal culture condition

**Fig. 2:**
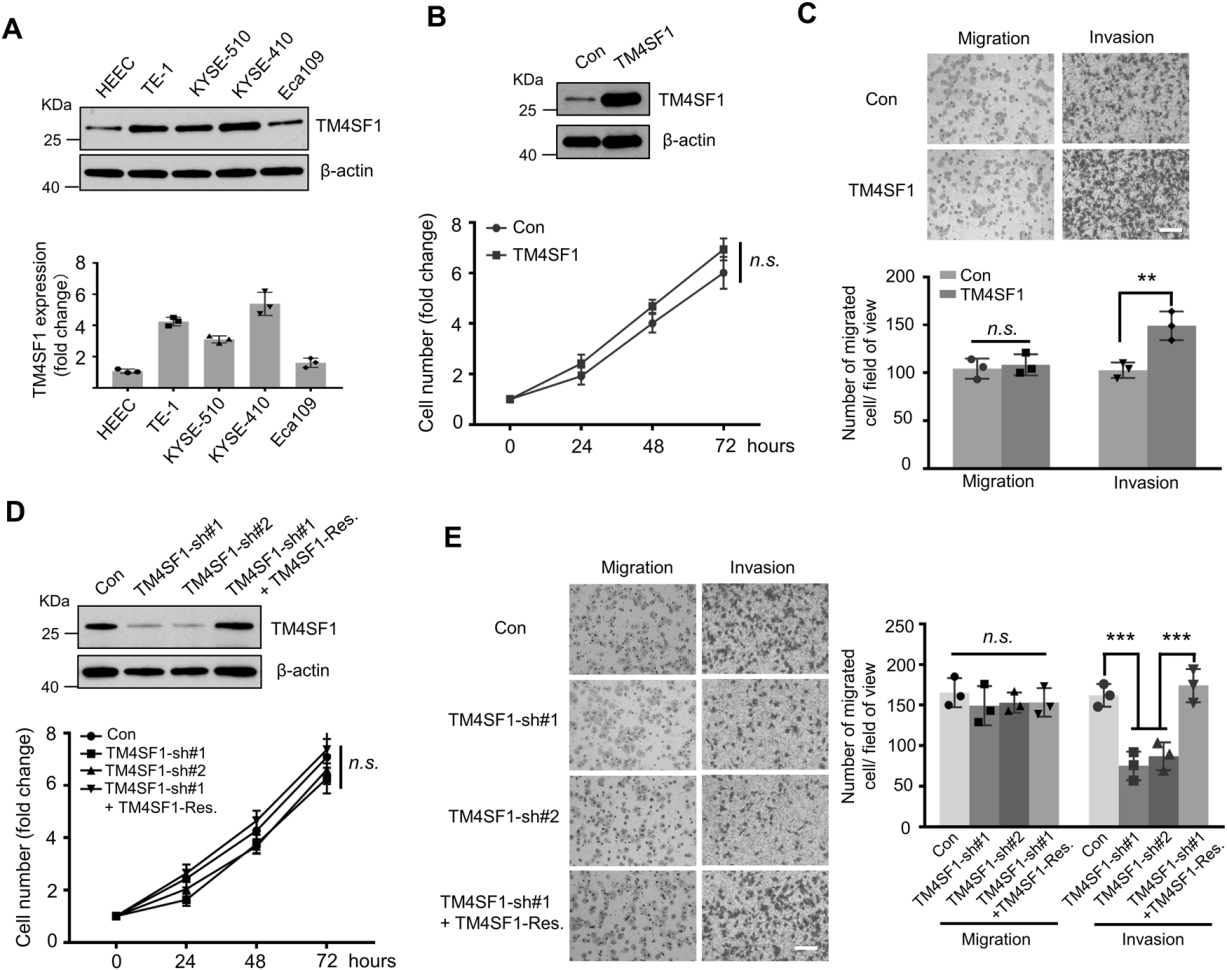
The effects of TM4SF1 on cell proliferation, migration, and invasion in related ESCC cells. **A** Lysates from indicated ESCC cells were immunoblotted by anti-TM4SF1 antibody, β-actin was used as a loading control (upper panel). Quantitative data was determined by a two-tailed unpaired *t* test from 3 independent experiments (lower panel), Error bars are means ± s.d. **B**, **D** Lysates from control (Con) and TM4SF1 stably overexpressed (TM4SF1-OE) Eca109 cells (**B**), and Con, TM4SF1-knockdown (KD) KYSE-410 cells, and KD cells transduced with TM4SF1 (**D**) were immunoblotted by anti-TM4SF1 antibody, β-actin was used as a loading control (upper panel). Indicated cells were starved with serum-free RPMI 1640 for 24 h and then released with RPMI 1640 containing 10% FBS; the numbers of live cells were counted at the indicated time points. Cell numbers were normalized to those at 0 h. Statistical significance was determined by a two-tailed unpaired *t* test. Error bars are means ± s.d. (lower panel; *n* = 3; *n.s*., not statistically significant). **C**, **E** The migration and invasion abilities of indicated Eca109 (**C**) and KYSE-410 (**E**) cells were analyzed by transwell assay. The representative images were recorded by phase-contrast microscopy. The quantitative number of cells was obtained from 3 independent experiments. The *P* values were determined by two-tail unpaired *t* test (n.s., not statistically significant; **P < 0.01; ***P < 0.001 by two-tail unpaired *t* test). Error bars are means ± s.d. Scale bar, 250 µm.

To further explore the functional significance of TM4SF1 in ESCC, we first determined TM4SF1 protein levels in a panel of human ESCC cell lines. As shown in Fig. 2A, TM4SF1was upregulated in all 4 analyzed ESCC cell lines (TE-1, KYSE-510, KYSE-410, and Eca109) compared to it in primary human endometrial epithelial cells (HEEC). We then established the TM4SF1-overexpression (OE) Eca109 cells to explore the role of TM4SF1 in multiple cell behaviors, including cell proliferation, migration, and invasion. The overexpression efficiency was confirmed by western blot (Fig. 2B, upper panel), and the cell proliferation was not affected by TM4SF1 (Fig. 2B, lower panel). Interestingly, growth factor reduced (GFR) basement membrane matrigel coating mediated Boyden chamber analysis showed that overexpression of TM4SF1 led to a significant increase in cell invasion, but not migration, in Eca109 cells under normal culture condition (Fig. 2C). Conversely, shRNA-mediated knockdown of TM4SF1 in KYSE-410 cells decreased cell invasion capability, but not cell proliferation and migration capabilities, under normal culture condition (Fig. 2D, E); this inhibitory effect on cell invasive could be reversed by restoration of TM4SF1 (Fig. 2E). These results suggest that TM4SF1 positively regulates cell invasion, but not migration and proliferation, in ESCC cells under normal culture condition.

### TM4SF1 promotes ESCC cell migration in a laminin-dependent manner

**Fig. 3:**
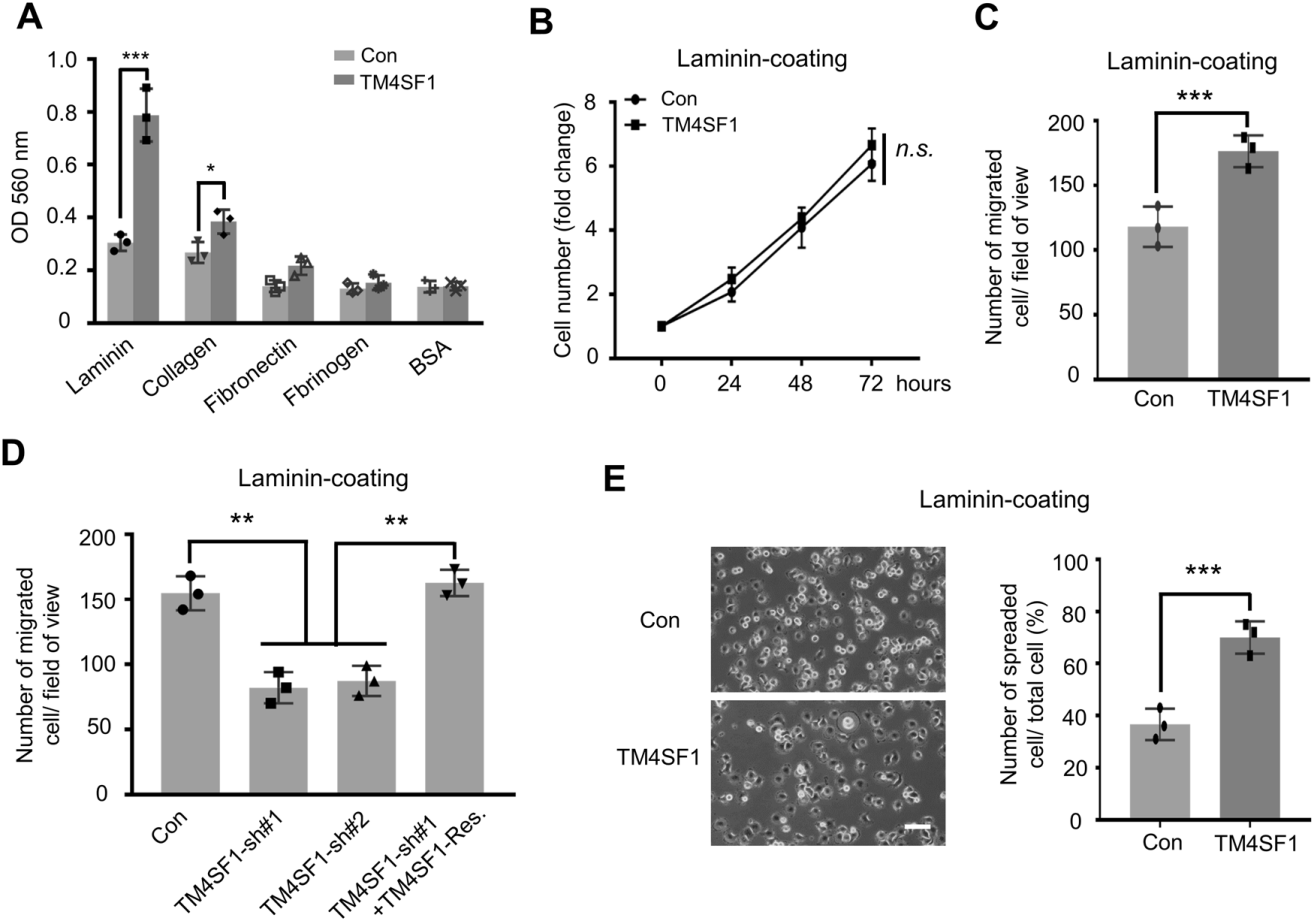
TM4SF1 promotes laminin-mediated cell adhesion, migration, and spreading in ESCC cells. **A** Adhesion ability of Con and TM4SF1-OE Eca109 cells upon various ECM proteins. Cell suspensions were planted on the different ECM (laminin, collagen, fibronectin, and fibrinogen)-coated plates for 40 min at 37 °C. BSA was used as a control. Attached cells were stained and checked by colorimetric detection. The quantitative data were determined by a two-tailed unpaired *t* test. Error bars are means ± s.d. (*n* = 3; ***P < 0.001; *P < 0.05 by two-tail unpaired *t* test). **B** Indicated Eca109 cells were cultured on the laminin-coated dishes, starved with serum-free RPMI 1640 for 24 h and then released with RPMI 1640 containing 10% FBS, the numbers of live cells were counted at the indicated times. Cell numbers were normalized to those at 0 h. Statistical significance was determined by a two-tailed unpaired *t* test. Error bars are means ± s.d. (*n* = 3; n.s., not statistically significant). **C**, **D** The migration ability of indicated Eca109 (**C**) and KYSE-410 (**D**) cells was determined by transwell assay on laminin-coated condition. Representative photos were taken and then the migrated cells were counted. Statistical significance was determined by a two-tailed unpaired *t* test. Error bars are means ± s.d. (*n* = 3; ***P < 0.001; **P < 0.01). **E** Indicated Eca109 cells were detached, suspended in serum-free RPMI 1640 for 1 h, and then plated on the laminin-precoated (10 µg/ml) dishes. After incubation for 40 min, spread cells were fixed with PFA, and the representative photos were taken (left panel). The percentages of adherent cells were determined by a two-tailed unpaired *t* test. Error bars are means ± s.d. (right panel; *n* = 3; ***P < 0.001). Scale bar, 50 µm.

The different effects of TM4SF1 on cell migration and invasion encourage us to explore the underlying mechanisms involved in TM4SF1-mediated invasion. Considering ECM proteins were major components of the GFR basement membrane matrigel, which we used to coat the Boyden chamber for the invasion assay (Fig. 2C, E), we detected the ECM binding profiles of Con and TM4SF1-OE Eca109 cells. Intriguingly, compared with Con cells, TM4SF1-OE cells attached more strongly to laminin (Fig. 3A), one major ECM component of matrigel, suggesting that the increased invasion ability of TM4SF1-OE cells may be due to the enhancement of the adhesiveness to laminin.

It is well known that laminin plays essential roles in multiple tumor biological functions [21], so we questioned whether laminin was also involved in regulating TM4SF1 in cell proliferation and migration. Interestingly, under laminin-coating condition, the Con and TM4SF1-OE Eca109 cells exhibited comparable proliferation ability (Fig. 3B). However, the migration ability of TM4SF1-OE cells was significantly increased when compared to the Con group (Fig. 3C), highlighting the importance of laminin in TM4SF1-mediated cell migration. As expected, shRNA-mediated knockdown of TM4SF1 in KYSE-410 cell line decreased the capability of cell migration under laminin-coating condition, and this inhibitory effect could be reversed by restoration of TM4SF1 (Fig. 3D). Considering adhesive interactions play fundamental roles in cell migration, we compared the cell spreading abilities of Con and TM4SF1-OE Eca109 cells. As shown in Fig. 3E, the TM4SF1-OE cells exhibit enhanced spreading ability on laminin when compared with Con ones. These findings indicate that TM4SF1 promotes explicitly cell adhesion, spreading, migration, and invasion in a laminin-dependent manner.

### TM4SF1 promotes laminin-mediated cell migration by interacting with integrin α6

**Fig. 4:**
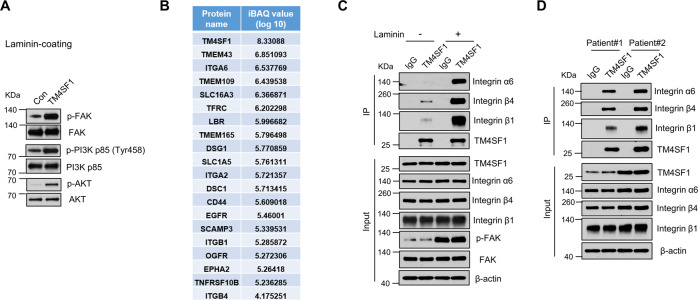
Laminin promotes the interaction between TM4SF1 and integrin α6 in Eca109 cells. **A** Con- and TM4SF1 overexpressed- Eca109 cell lysates (under laminin-coating condition) were immunoblotted by anti-p-FAK, anti-FAK, anti-p-PI3K p85 (Try458), anti- PI3K p85, anti-pAKT, and anti-AKT antibodies. **B** List of TM4SF1-associated cell membrane proteins in Eca109 cells under laminin-coating condition, identified by mass spectrometric analysis. **C** Co-IP of endogenous TM4SF1 with endogenous integrin α6, β4, and β1 in cell lysate. TM4SF1 was immunoprecipitated from Eca109 cells, which were cultured on the dishes with or without laminin-coating, and immunoblotted with antibodies against integrin α6, β4, β1, and TM4SF1. The input was immunoblotted with antibodies against integrin α6, β4, β1, p-FAK, FAK, TM4SF1, and β-actin. **D** Co-IP of endogenous TM4SF1 with endogenous integrin α6, β4, and β1 in ESCC patient tissues. TM4SF1 was immunoprecipitated from homogenized ESCC patient tissues lysate and immunoblotted with antibodies against integrin α6, β4, β1, and TM4SF1.

Next, we wonder about the underlying mechanisms involved in the laminin/TM4SF1-mediated ESCC cell migration. It is well known that FAK is a critical molecule involved in focal adhesion formation via tyrosine phosphorylation during the cell adhesion process, which can facilitate intracellular signaling events [22]. We first examined whether the FAK signaling pathway was aberrantly activated in TM4SF1-OE cells under laminin coating. As shown in Fig. 4A, the phosphorylated levels of FAK, PI3K p85, and AKT were remarkably increased in TM4SF1-OE cells as compared to Con groups, indicating that activation of FAK/PI3K/AKT signaling pathway may be required for the pro-migration effect of TM4SF1 on laminin coating condition.

To investigate the molecular mechanism by which TM4SF1 regulates cell migration, we attempted to identify TM4SF1-interacting membrane proteins upon laminin coating by using V5-tagged TM4SF1. We performed the immunoprecipitation (IP), followed by mass spectrometric (MS) analysis, which identified a list of TM4SF1-interacting membrane proteins; among these proteins, integrins, as potential laminin-binding receptors and FAK signaling regulators, have garnered our attention (Fig. 4B). Specifically, these integrins were three previously unreported TM4SF1-interacting integrins, including α6, α2, and β4, as well as one reported integrin β1, among which integrin α6 was ranked the top one according to the intensity-based absolute quantitation (iBAQ) value (Fig. 4B). Considering integrin α6β4 and α6β1 are two main members of laminin receptors family, which mediate cell adhesion, and transmit mechanical and laminin-specific signals to the cell interior [23, 24], it is reasonable to speculate that the interaction between TM4SF1 and integrin α6 is mediated by laminin. The endogenous co-IP assays showed that laminin-coating significantly enhanced the interaction of TM4SF1 with integrin α6, β4, and β1 in Eca109 cells (Fig. 4C). Notably, the interaction between TM4SF1 and integrin α6, β4, and β1 can also be validated in ESCC patient tissues (Fig. 4D). Taken together, these results suggest that TM4SF1-integrin α6 complex formation may be crucial for FAK signaling activation under laminin-coating condition.

### TM4SF1/integrin α6/FAK signaling axis promotes laminin-mediated ESCC cell migration

**Fig. 5:**
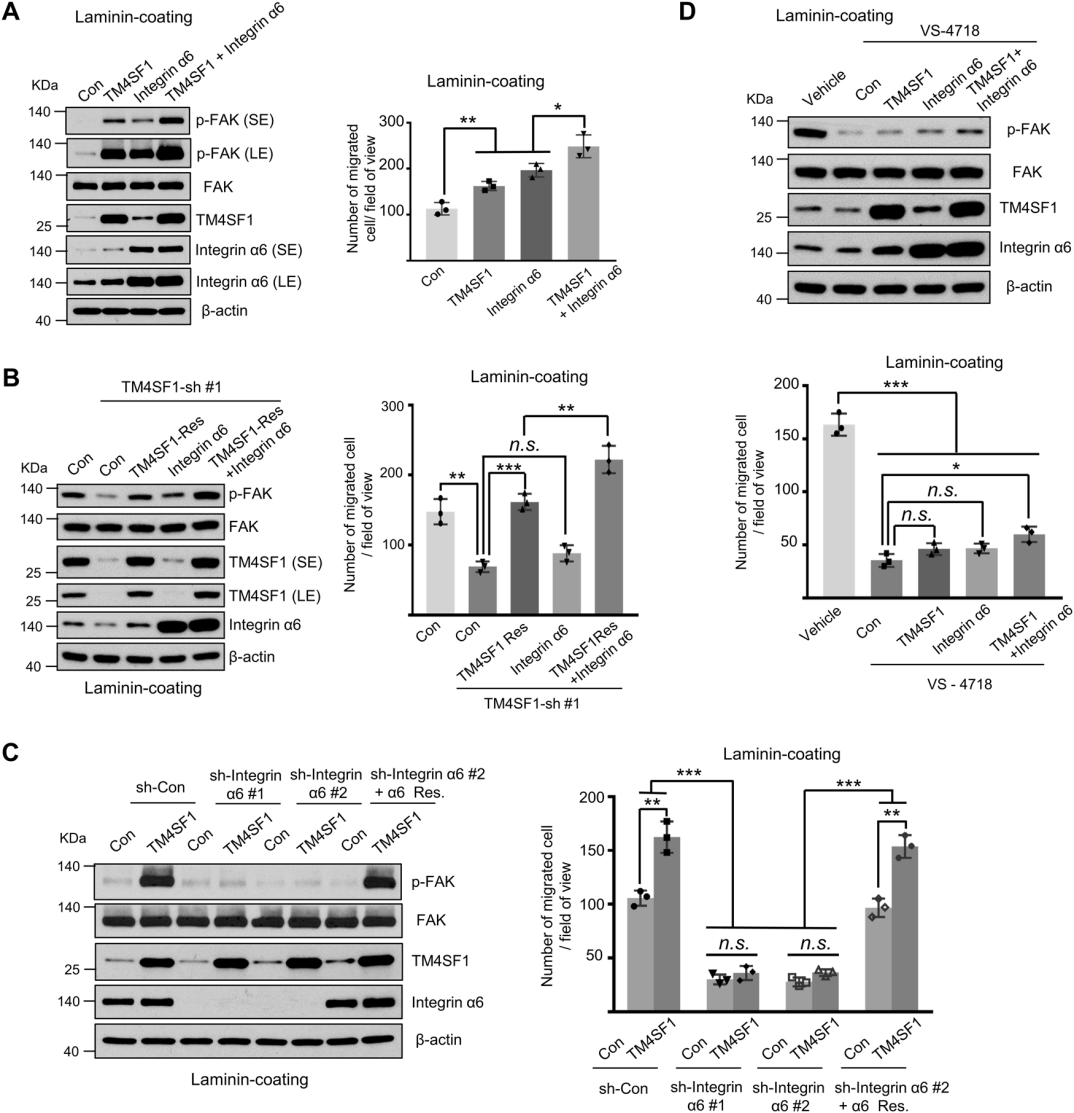
The Laminin-TM4SF1-integrin α6-FAK signaling axis mediates ESCC cell migration. **A**–**C** Immunoblotting of p-FAK, FAK, TM4SF1, integrin α6, and β-actin in (left panel) and migration ability of (right panel) TM4SF1-overexpressed, integrin α6-overexpressed, and TM4SF1/integrin α6 double overexpressed Eca109 cells (**A**), Con-, TM4SF1-knockdown-, related TM4SF1 rescued-, integrin α6- overexpressed, and TM4SF1-rescued/integrin α6- overexpressed KYSE-410 cells (**B**), and integrin α6-knockdown- and related α6 rescued- Con or TM4SF1 overexpressed Eca109 cells (**C**) under laminin coating condition (LE: long exposure; SE: short exposure). Representative photos were taken, and then the migrated cells were counted. Statistical significance was determined by a two-tailed unpaired *t* test. Error bars are means ± s.d. (right panel; *n* = 3; *P < 0.05; **P < 0.01; ***P < 0.001). **D** Immunoblotting of p-FAK, FAK, TM4SF1, integrin α6, and β-actin in (upper panel) and migration ability of (lower panel) TM4SF1-overexpressed, integrin α6-overexpressed, and TM4SF1/integrin α6 double overexpressed KYSE-410 cells pre-treated with or without VS-4718 (1 μM) for 24 h. Representative photos were taken and then the migrated cells were counted. Statistical significance was determined by a two-tailed unpaired *t* test. Error bars are means ± s.d. (right panel; *n* = 3; n.s., not statistically significant; *P < 0.05; ***P < 0.001).

The data provided above led us to speculate that the TM4SF1-integrin α6 complex may affect FAK signaling, thereby contributing to laminin-dependent cell migration. To address this, we first try to understand the role of integrin α6 in laminin/TM4SF1-mediated cell signaling and migration. We overexpressed the integrin α6 in Con or TM4SF1-OE Eca109 cells. As shown in Fig. 5A, under laminin-coating condition, compared to the Con group, the expression level of FAK phosphorylation (p-FAK) was increased in integrin α6-OE cells and was further enhanced in TM4SF1 and integrin α6-double OE cells; meanwhile, the abilities of cell migration were also remarkably increased after TM4SF1-integrin α6 overexpression. To confirm the results, we knocked down TM4SF1 and then rescued TM4SF1, or integrin α6, or both genes in KYSE-410 cells. As shown in Fig. 5B, p-FAK and cell migration on laminin exhibited a significant increase in TM4SF1-integrin α6 double rescue group compared with the individual overexpression of TM4SF1 or integrin α6. In addition, the increased expression level of p-FAK in TM4SF1-OE Eca109 cell was largely canceled in shRNA-mediated integrin α6 knockdown groups, as well as the migration ability, and this inhibitory effect on both p-FAK expression and cell migration could be reversed by restoration of integrin α6 under laminin-coating condition (Fig. 5C). These results clearly show that the integrin α6 is important for TM4SF1/laminin-mediated FAK signaling and migration in ESCC cells.

To investigate whether FAK is a central mediator of TM4SF1-integrin α6 mediated cell signaling, we next treated the cells with pharmacologic inhibitors. As shown in Fig. 5D, VS-4718 (a selective bispecific inhibitor for FAK and Pyk2 kinases) eliminated TM4SF1-integrin α6 induced signal transduction and migration of KYSE-410 cells. These results indicate that TM4SF1-integrin α6 complex is upstream of FAK activation on laminin coating condition, highlighting the importance of TM4SF1/integrin α6/FAK signaling axis in laminin-mediated ESCC cell migration.

### TM4SF1/integrin α6/FAK signaling axis promotes ESCC metastasis

**Fig. 6:**
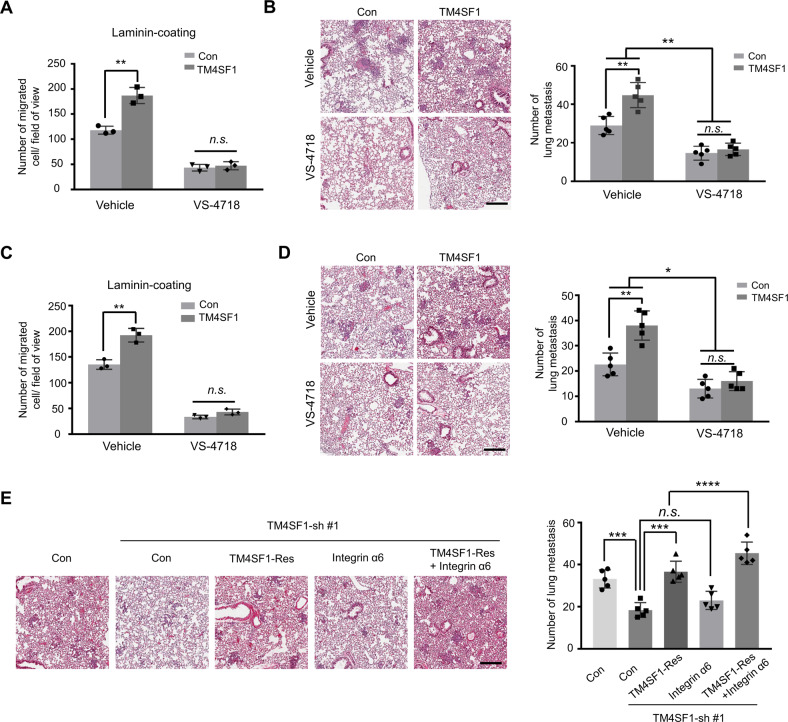
TM4SF1/integrin α6/FAK signaling axis promotes ESCC metastasis. **A**, **C** TM4SF1 overexpressed Eca109 (**A**) and KYSE-510 (**C**) cells were treated with or without VS-4718 (1 μM) for 24 h, the migration ability was further determined by transwell assay on laminin-coating condition. The number of migrated cells were counted. Statistical significance was determined by a two-tailed unpaired *t* test. Error bars are means ± s.d. (*n* = 3; n.s., not statistically significant; **P < 0.01). **B**, **D** The representative images (left panels) and number (right panels) of lung metastatic nodules of BALB/c-nude mice with tail vein injection of Eca109-Con and Eca109-TM4SF1-OE cells (**B**) and KYSE-510 -Con and KYSE-510 -TM4SF1-OE cells (**D**) are presented. Scale bar, 250 µm. Mice were administered with or without VS-4718 (50 mg/kg) by oral gavage two times a day. The numbers of lung metastasis were statistically analyzed by a two-tailed unpaired *t* test. Error bars are means ± s.d. (*n* = 5; n.s., not statistically significant; *P < 0.05; **P < 0.01). **E** The representative images (left panel) and number (right panel) of lung metastatic nodules of BALB/c-nude mice with tail vein injection of Con-, TM4SF1-knockdown-, related TM4SF1 rescued-, integrin α6- overexpressed, and TM4SF1-rescued/integrin α6- overexpressed KYSE-410 cells are presented. Scale bar, 250 µm. The numbers of lung metastasis were statistically analyzed by a two-tailed unpaired *t* test. Error bars are means ± s.d. (*n* = 5; n.s., not statistically sig*n*ificant; ***P < 0.001; ****P < 0.0001).

The data described above led us to investigate whether the TM4SF1/integrin α6/FAK signaling axis also contribute to ESCC metastasis. As shown in Fig. 6A, we first found that VS-4718 treatment eliminated the difference between Con and TM4SF1-OE Eca109 cells for cell migration, indicating that the FAK pathway is also responsible for TM4SF1-mediated cell migration under laminin-coating condition in Eca109 cells.

Next, we examined the effect of TM4SF1 on tumor metastasis in a mouse lung colonization model. We checked the metastatic nodules in the lungs after intravenous injection of Con and TM4SF1-OE Eca109 cells via tail vein. As shown in Fig. 6B, the number of pulmonary metastatic nodules was evidently increased in the TM4SF1-OE group compared to the Con group. However, blockade of the FAK signal by VS-4718 treatment largely eliminated the prometastatic function of TM4SF1 on lung metastatic colonization (Fig. 6B). Importantly, these phenotypes can also be further validated in KYSE-510 cells (Fig. 6C, D). These results demonstrate that TM4SF1 promotes ESCC cell migration and metastasis via FAK signaling.

To further examine the importance of TM4SF1/integrin α6/FAK signaling axis on ESCC metastasis, nude mice were also intravenously injected with Con, TM4SF1-knockdown or related TM4SF1-rescue, integrin α6-overexpression, and TM4SF1-rescue plus integrin α6-overexpression KYSE-410 cells via tail veins. As shown in Fig. 6E, the number of pulmonary metastatic nodules formed by TM4SF1-knockdown KYSE-410 cells was significantly fewer than the Con cells, whereas restoration of TM4SF1, but not overexpress integrin α6, resulted in comparable number of metastatic nodules compared with the Con one. Moreover, overexpress integrin α6 in TM4SF1-rescued cells further increased the metastatic nodule numbers when compared with only TM4SF1-rescued group (Fig. 6E). Taken together, these results indicate that TM4SF1 is essential for integrin α6-mediated metastasis, further highlighting the importance of the TM4SF1-integrin α6 complex in ESCC metastasis.

### TM4SF1/integrin α6/FAK signaling axis positively correlates with ESCC progression and survival

**Fig. 7:**
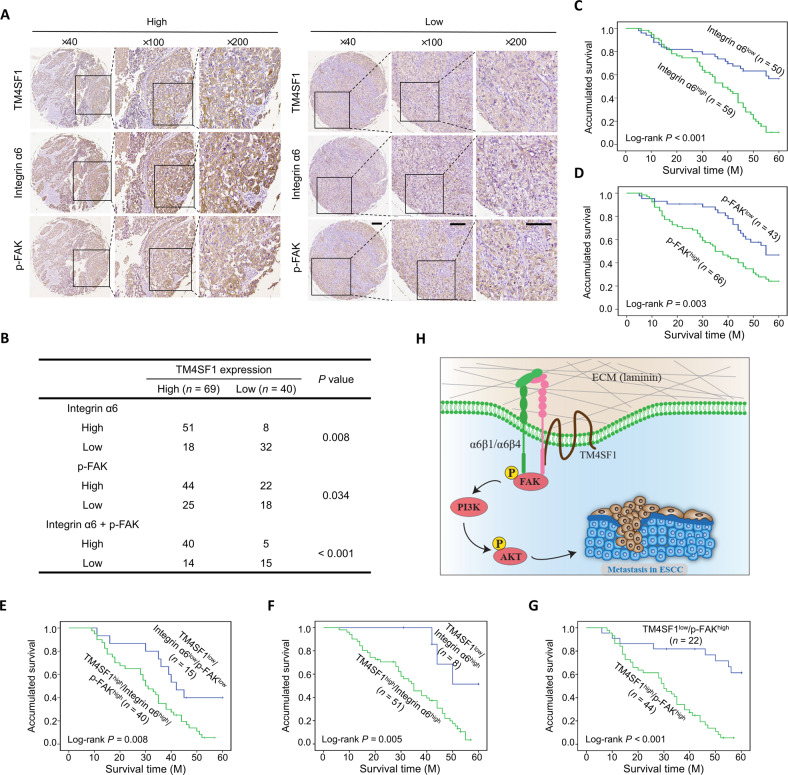
TM4SF1-integrin α6-FAK signaling axis is positively related to ESCC. **A** Immunohistochemical stainings of TM4SF1, integrin α6, and p-FAK in representative ESCC tissue microarray (*n* = 109). Score = 0–3 (low) and score = 4–12 (high) indicate TM4SF1, integrin α6, and p-FAK levels in representative tumor tissues. Scale bar, 200 µm. **B** Correlation between TM4SF1, integrin α6, and p-FAK protein levels in patients with ESCC. The *P* value was calculated from a χ^2^ test. (C-G) Kaplan–Meier analysis of overall survival in a cohort of ESCC patients (*n* = 109), stratified by the protein level of integrin α6 (**C**), p-FAK (**D**), low expression level of TM4SF1/integrin α6 /p-FAK and high expression level of TM4SF1/integrin α6/p-FAK (**E**), low expression level of TM4SF1/high expression level of integrin α6 and high expression level of TM4SF1/integrin α6 (**F**), or low expression level of TM4SF1/high expression level of p-FAK and high expression level of TM4SF1/p-FAK (**G**). Statistical significance was determined by a log-rank test, *P* < 0.001 (**C**), *P* = 0.003 (**D**), *P* = 0.008 (**E**), *P* = 0.005 (**F**), or *P* < 0.001 (**G**). **H** Working model for the role of TM4SF1 in regulating laminin-mediated signaling and metastasis.

To address whether the TM4SF1/integrin α6/FAK signaling axis is relevant in ESCC tissues, we examined the expression of TM4SF1, integrin α6, and p-FAK through IHC staining in TMA samples (*n* = 109). According to the immunostaining scores, these samples were divided into high (score 4–12) and low (score 0–3) levels of TM4SF1, integrin α6, and p-FAK groups. Representative images of IHC staining with different intensities of TM4SF1, integrin α6, and p-FAK in the same region are shown in Fig. 7A. Strikingly, TM4SF1 expression was positively correlated with integrin α6 and p-FAK expression (Fig. 7A). The detailed expression patterns of TM4SF1, integrin α6, and p-FAK were summarized in Fig. 7B as follows: 29.4% (32 of 109) of the tumors with low TM4SF1 expression exhibited low integrin α6 expression (*P* = 0.008), 16.5% (18 of 109) of the tumors with low TM4SF1 expression exhibited low p-FAK expression (*P* = 0.034), and 14% (15 of 109) of the tumors with low TM4SF1 expression exhibited both low integrin α6 and p-FAK expression (*P* < 0.001); meanwhile, 46.8% (51 of 109) of the tumors with high TM4SF1 expression showed high integrin α6 expression (*P* = 0.008), 40.4% (44 of 109) of the tumors with high TM4SF1 expression showed high p-FAK expression (*P* = 0.034), and 37% (40 of 109) of the tumors with high TM4SF1 expression exhibited both high integrin α6 and p-FAK expression (*P* < 0.001).

Moreover, high expression of integrin α6 and p-FAK were significantly associated with poor outcomes in ESCC (Fig. 7C, D), and patients classified as TM4SF1^low^/integrin α6^low^/p-FAK^low^ showed better disease outcome than the TM4SF1^high^/integrin α6^high^/p-FAK^high^ group (Fig. 7E). Notably, the Kaplan-Meier survival curves also showed that the overall survival of patient classified as TM4SF1^high^/integrin α6^high^ or TM4SF1^high^/pFAK^high^ group had a poorer outcome than TM4SF1^low^/integrin α6^high^ or TM4SF1^low^/pFAK^high^ group, respectively, further suggesting the importance of TM4SF1/integrin α6/FAK axis in ESCC (Fig. 7F, G). Our results indicate that TM4SF1/integrin α6/FAK axis is positively correlated with tumor progression and poor prognosis and has the potential to serve as a prognostic factor and therapeutic target in ESCC.

## Discussion

The L6 protein TM4SF1, as a critical signal transducer is overexpressed in most malignant tumors and involved in the progression and migration. In the present study, we outline the novel underlying mechanism responsible for the pro-migratory role of TM4SF1 in ESCC and provide new insights into the role of ECM and TEMs in the regulation of cellular signaling. In detail, the expressions of TM4SF1 were strongly upregulated in ESCC tissues than in non-tumor tissues at both mRNA and protein levels. TM4SF1 mediated ESCC cell migration and invasion in a laminin-dependent manner by interacting with integrin α6. Further study showed that the TM4SF1-integrin α6 complex promotes ESCC cell migration and metastasis via FAK/PI3K/AKT signaling (Fig. 7H). Moreover, TM4SF1 expression was positively correlated with integrin α6 and p-FAK expression, and activated TM4SF1/integrin α6/FAK signaling axis is correlated with poor survival in ESCC patients.

Several reports have indicated that the tetraspanin superfamily, including TM4SF1, could form TEMs through their interaction with other proteins, which play important roles in modulating cell adhesion, propagation, migration, and invasion in multiple cancers, including human lung, breast, prostate, and pancreatic cancer [10]. It is worth noting that TEMs can influence cell migration via regulating the surface expression patterns of relative components of these functional complexes and intracellular signaling [9]. For example, TM4SF1 couples the collagen receptor tyrosine kinase DDR1 to PKC α and augments JAK-STAT signaling, promoting multi-organ site metastatic reactivation [11]. Besides, TM4SF1 can interact with integrin α5β1 via its EC2 domain, and this complex is critical for integrin-mediated cell adhesion to ECM, which further activates PI3K/AKT/mTOR signaling pathway [10, 25]. Our study demonstrated that TM4SF1 was dramatically upregulated in ESCC tissue samples and positively correlated with poor prognosis. Mechanically, TM4SF1 promoted ESCC cell migration and metastasis in a laminin-dependent manner. Considering a growing body of evidence for the TEMs formed by TM4SF1 and other proteins during migration, and also our MS analysis identified a series of TM4SF1-interacting integrins, it is reasonable to speculate that TM4SF1 may be associated with laminin receptor integrins which facilitate cell adhesion and motility by activating downstream signal pathways in ESCC cells. Consistent with our hypothesis, we identified and validated the interaction between TM4SF1 and integrin α6 by endogenous co-IP assays in Eca109 cells upon laminin treatment. These findings indicated that TM4SF1 could associate with specific integrins in cancer/cell-type dependent manner. Of note, Xue et al. show that TM4SF1 is essential for the migration and invasion in KYSE-150 and KYSE-180 ESCC cell lines [12]. Based on our results, one possibility may be that the expression levels of TM4SF1-interacted integrins and the membrane receptors that TM4SF1 interacted with are variable in a cancer or ESCC cell type-dependent manner, thus mediating different downstream signal pathways, which need further investigation.

Importantly, Ma et al. demonstrate that integrin α6 promotes ESCC metastasis through miR-92b/integrin α6/AKT axis signal pathway, however, the detailed mechanisms remain unknown [17]. Here, we found that the TM4SF1-integrin α6 complex was essential for ESCC migration and metastasis, highlighting the importance of the cross-talk between TM4SF1 and integrin α6. Of course, we could not exclude the roles of other member receptors, especially the receptor tyrosine kinases (RTKs) [24, 26], in integrin α6β4-mediated cellular signaling, since our group have reported that integrin α6β4 closely associates with EGFR, which can also activate ERK and AKT [27–29]. Further studies are also needed to investigate the cross-talks between integrin α6 and RTKs during the development and progression of ESCC.

In fact, laminin contains at least 16 different isoforms. However, it is well known that laminin-5 (also named laminin-332), as the preferred ligand of integrin α6β4 and α6β1 [30, 31], can promote the invasion of ESCC cells [32]; further investigations are needed to identify the exact laminin isoform involved in TM4SF1/integrin α6-mediated metastasis and the mechanistic roles of TM4SF1-containing (TEMs) in ESCC in more detail. In addition to laminin, the GFR basement membrane matrix also contains limited growth factors, such as transforming growth factor (TGF-β), epidermal growth factor (EGF), insulin-like growth factor (IGF-1), and basic fibroblast growth factor (bFGF), the effect of these growth factors on TM4SF1-mediated cell migration will also be an exciting part, which will present a more comprehensive picture of TM4SF1’s function in tumor microenvironment.

Consistent with the importance of TM4SF1 in ESCC cells we observed, our TMA analysis indicated that the upregulated TM4SF1 was closely associated with worse clinical outcomes and shorter survival in ESCC patients, suggesting that TM4SF1 may serve as a potential prognostic biomarker for predicting metastasis and a novel therapeutic target in ESCC. It is worthy of note that the role of TM4SF1 in cancer remains controversial [7, 33–36]. High expression of TM4SF1 predicts a poor prognosis in many types of cancer, such as glioma, colorectal cancer, breast cancer, and ovarian cancer, but predicts a good prognosis in gastric cancer, pancreatic cancer, breast cancer, and malignant pleural mesothelioma [10]. Here our study, for the first time, provides the evidence that TM4SF1 is positively correlated with TNM stage, N classification, differentiation, tumor size, and poor outcome. However, T classification, one of the cancer malignancy risk factors, showed no significant relation with TM4SF1 expression statistically, further study are needed by expanding the sample size.

In conclusion, our study demonstrates for the first time that TM4SF1 can promote laminin-mediated migratory and metastatic ability by interacting with integrin α6 via FAK/PI3K/AKT signaling, which provides new insights into the mechanistic roles of TM4SF1 in ESCC.

## Materials and methods

### Specimens and immunohistochemistry (IHC)

The cancer tissues (T) and paired adjacent tissues (N) were obtained from Affiliated Hospital of Yangzhou University (Yangzhou University, Yangzhou, China) from 2012 to 2013. None of the patients received any radiochemotherapy before the operation. IHC staining was performed as described previously [37]. Whole-slide images were captured using an Aperio CS2 slide scanner system (Leica Biosystems, Wetzlar, Germany) at a ×40 magnification. IHC stainings of TM4SF1, integrin α6, and p-FAK were analyzed by using the immunoreactive score (IRS) system. The percentage of positive cells was scored on a scale of 0 to 4: 0 if 0% of tumor cells were positive, 1 if 1–25% of cells were positive, 2 if 26–50% were positive, 3 if 51–75% were positive, and 4 if 76–100% were positive. Staining intensity was scored on a scale of 0 to 3 (3 is the strongest). Final IRS score = (score of the staining intensity) × (score of the percentage of positive cells). Score = 0–3 was considered low expression and score = 4–12 was considered high expression. Furthermore, the study was carried out in accordance with the approved guidelines according to the Ethics Committee at Affiliated Hospital of Yangzhou University (Yangzhou University, Yangzhou).

### Online database analysis

The mRNA levels of TM4SF1 in 161 EC tissues and 418 normal tissues were compared using the TNMplot online database (https://www.tnmplot.com/) as described before [20].

### Antibodies and reagents

The experiments were performed using the following antibodies: Rabbit monoclonal antibodies (mAbs) to AKT (1:1000, Catalog #9272S), p-AKT (1:1000, Catalog #4060S), p-PI3K p85 (Tyr458) (1:1000, Catalog #17366S), PI3K p85 (1:1000, Catalog #4257S), integrin α6 (1:1000, Catalog #3750S), p-FAK (1:1000, Catalog #8556S), and integrin β4 (1:1000, Catalog #14803S) were from Cell Signaling Technology (Massachusetts, USA), anti-TM4SF1 antibodies used for WB and IHC were from R&D Systems (Catalog #AF7514, Minnesota, USA) and Novus Biologicals (Catalog #NBP1-76549, Colorado, USA), respectively; Mouse mAbs against FAK (1:1000, Catalog #610088) and integrin β1(1:2000, Catalog #610467) were from BD Biosciences (New Jersey, USA), glyceraldehyde-3-phosphate dehydrogenase (GAPDH, 1:1000, Catalog #sc-365062) and β-actin (1:1000, Catalog #sc-47778) were purchased from Santa Cruz Biotechnology (Texas, USA). The integrin α6 and p-FAK antibodies for IHC was purchased from Proteintech (Catalog #27189-1-AP, Illinois, USA) and Thermo Fisher Scientific (Catalog #700255, Massachusetts, USA), respectively. Laminin, collagen, Fibronectin (FN), and Fbrinogen were from Sigma (Massachusetts, USA). The growth factor reduced (GFR) basement membrane matrix was purchased from Corning (New York, USA). IgG was purchased from Bio-Rad Laboratories (California, USA). Protein-A/G PLUS beads were purchased from Santa Cruz Biotechnology (Texas, USA). Anti-V5-tag mAb-magnetic beads were purchased from Medical & Biological Laboratories (Aichi, Japan). The PND-1186 (VS-4718) was purchased from Selleck Chemicals (Texas, USA). iScript complementary DNA (cDNA) Synthesis Kit and iTaq Universal SYBR Green Kit were from Bio-Rad Laboratories (California, USA). TRIzol reagent was obtained from Invitrogen (Massachusetts, USA).

### Cell lines and cell culture

Human esophageal epithelial cells (HEEC), human ESCC cell lines (Eca109 and TE-1), and HEK293T cells were purchased from the Cell Bank of the Chinese Academy of Sciences (Shanghai, China). HEK293T cells was maintained at 37 °C in DMEM medium and other cell lines (HEEC, Eca109, TE-1, KYSE-510, and KYSE-410) were maintained at 37 °C in RPMI 1640 medium, supplemented with 10% fetal bovine serum (FBS), under a humidified atmosphere containing 5% CO_2_, except for the virus production.

### Overexpression and knockdown vectors of TM4SF1 and integrin α6

The cDNA of the human TM4SF1 and integrin α6 were amplified by PCR from the reverse-transcribed product of Eca109 cell total RNA and then subcloned into the pDONR201 vector as described previously [38]. The resultant cDNAs were confirmed by sequence. The cDNAs of TM4SF1 and integrin α6 from the pDONR201 were transferred into pLenti-CMV-Puro DEST (w118-1) (Addgene, #17452), pLenti-CMV-Hygro DEST (w117-1) (Addgene, #17454), or pLX304 (Addgene, #25890) vectors via LR reaction (GatewayTM cloning system kit, Invitrogen, Thermo Fisher Scientific, Massachusetts, USA).

For expressing short hairpin RNA, we used the pLKO.1-puro lentiviral vector. Inserted oligonucleotide sequences were listed as follows: shRNA#1 against TM4SF1, 5′-CCGGGTTTCCCATTCATACACCTATCTCGAGATAGGTGTATGAATGGGAAACTTTTT-3′, shRNA#2 against TM4SF1, 5′-CCGGTCAAGTAATAAATGGAGTGCTCTCGAGAGCACTCCATTTATTACTTGATTTTT-3′, shRNA#1 against integrin α6, 5′-CCGGGACAACGTGATCCGGAAATATCTCGAGATATTTCCGGATCACGTTGTCTTTTTG-3′, and shRNA#2 against integrin α6, 5’- CCGGTTCTTTAACTGCCGTAATTTACTCGAGTAAATTACGGCAGTTAAAGAATTTTTG-3′.

### Virus production, infection, and stable cell lines construction

Virus production and infection were performed as described previously [39]. In brief, the lentivirus-based TM4SF1 or integrin α6 overexpression and pLKO.1-knockdown vectors were cotransfected with pCAG-HIVgp and pCMV-VSV-G-RSV-Rev into HEK293T cells. After transfection for 48 h, the lentivirus supernatants were collected. The indicated cells were infected with the resultant viral supernatant for 72 h and then selected by hygromycin B, puromycin, or blasticidin to get resistant cells against these antibiotics. Stable cell lines were used in subsequent studies.

### Western Blot (WB) and Immunoprecipitation (IP)

WB and IP were performed as described previously [39]. Briefly, for WB, the homogenized patient tissues and the indicated cells were lysed in the cell lysate (20 mM Tris-HCl pH 7.4, 150 mM NaCl, 1% Triton X-100) with protease and phosphatase inhibitors for 30 min. After centrifugation, the supernatant was collected and protein concentrations were determined using a BCA protein assay kit (Pierce, Rockford, Illinois, USA). Proteins were resolved by SDS/PAGE and transferred to a PVDF membrane (Millipore, Sigma, Massachusetts, USA). After transfer, membranes were blocked with 5% non-fat milk in Tris-buffered saline with 0.05% Tween-20 (TBST) and incubated with the primary antibody at 4 °C overnight, followed by incubation with the secondary antibody conjugated with horseradish peroxidase (HRP). The bands were visualized with enhanced chemiluminescence substrate (Pierce, Illinois, USA).

For IP, the homogenized patient samples and cells were lysed in 0.1% Triton TBS buffer (20 mM Tris-HCl pH 7.4, 150 mM NaCl) with protease and phosphatase inhibitors, and then centrifuged. The supernatants were pre-cleared with protein-A/G PLUS beads and IgG at 4 °C for 30 min, followed by incubation with an antibody against TM4SF1 or IgG at 4 °C for 1 h, and were then incubated with protein-A/G PLUS beads at 4 °C overnight. The beads were washed with TBS buffer 3 times and the immunoprecipitates were subjected to SDS-PAGE.

### Mass spectrometry analysis

To identify TM4SF1-interacting proteins in Eca109 cells, we performed the immunoprecipitation and mass spectrometry as follows. V5-tagged TM4SF1 stably overexpression Eca109 cells were cultured on laminin-coated dishes. For IP, a total of 3 15-cm dishes of cells were lysed in 0.1% Triton TBS buffer (20 mM Tris-HCl pH 7.4, 150 mM NaCl) containing protease inhibitors at 4 °C for 30 min. Crude lysates were cleared by centrifugation, and the supernatants were incubated with 100 µl anti-V5-tag mAb-magnetic beads at 4 °C for 2 h. The beads were washed three times with TBS buffer. The bound proteins were eluted by boiling in loading buffer, resolved by SDS-PAGE, visualized by Coomassie blue staining, and subjected to mass spectrometric analysis.

### Cell growth analysis

Indicated cells (3 × 10^4^) were cultured on none-coated or Laminin-coated 6-cm dishes overnight, starved with serum-free RPMI 1640 for 24 h. After starvation, the cells were supplied with RPMI 1640 containing 10% FBS. The photographs of the same areas on the cultured dishes were taken at the indicated times (0, 24, 48, and 72 h), and the cell numbers were counted. Cell numbers were normalized to those at 0 h and statistically analyzed.

### Migration and invasion assays

Migration and invasion were examined by Boyden chamber assay (Corning transwell cell culture inserts, 8.0-mm inserts; Corning, New York, USA). Migration assay was performed as described previously [40]. For migration assay on laminin, each transwell was precoated on the bottom side with 10 µg/ml laminin in PBS containing 0.1% BSA at 4 °C overnight and then blocked with 5% BSA in RPMI 1640 at 37 °C for 1 h. The invasion assay was similarly performed with a modified Boyden chamber whose upper chamber was coated with growth factor reduced (GFR) basement membrane matrigel (Corning, #354230, New York, USA), and the rest of the protocol was finished similarly as the migration assay.

### Cell adhesion and spreading assays

For cell adhesion assay, 48-well plates were coated with different ECM (10 µg/ml) in PBS at 4 °C overnight and then blocked with 1% bovine serum albumin (BSA) in RPMI 1640 medium for 1 h at 37 °C. Indicated cell suspensions were allowed to attach to different ECM-coated plates for 40 min at 37 °C. Adherent cells were stained, extracted, and then measured at 560 nm wavelength in a plate reader. Triplicate wells were used for each group, and the average of the results was calculated.

For cell spreading assay, indicated cells were detached and suspended in serum-free RPMI 1640 medium with 0.1% BSA at 6 × 10^4^ cells/ml. After 40 min incubation on Laminin-coated plates, non-adherent cells were gently removed by PBS, and the attached cells were fixed with 4% paraformaldehyde (PFA) in PBS. Images were then taken by phase-contrast microscopy.

### RNA isolation and qPCR

Total RNA from ESCC tissues was extracted using TRIzol reagent (Invitrogen, Massachusetts, USA) according to the manufacturer’s protocol. Then reverse transcribed with an iScript complementary DNA (cDNA) Synthesis Kit (Bio-Rad Laboratories, California, USA). First-strand cDNA was used for real-time PCR with the iTaq Universal SYBR Green Kit (Bio-Rad Laboratories, California, USA). The sequences of the primers were as follows: TM4SF1, forward, 5′-GGTTCTTTTCTGGCATCGTAGGAGGTG-3′, reverse, 5′-CTGGCCGAGGGAATCAAGACATAGTG-3′; GAPDH, forward, 5′-GGAGTCAACGGATTTGGT-3′, reverse, 5′-GTGATGGGATTTCCATTGAT-3′.

Real-time PCR and data collection were performed on the ABI PRISM 7700 Sequence Detector (Applied Biosystems, Thermo Fisher Scientific, Massachusetts, USA).

### Animal study

4 weeks old female BALB/c-nude mice were randomly assigned to different groups. A total of 1 × 10^6^ cells (Eca109-, KYSE-510-, and KYSE-410- related TM4SF1-overexpression or TM4SF1-knockdown/rescue cell lines) were injected via the tail vein of each mice. After 7 days, the mice for the overexpression experiment were further randomly divided into two groups (*n* = 8 for each group) and administered with or without VS-4718 (50 mg/kg) by oral gavage two times a day. On the last day of the treatment, mice were sacrificed and the lungs were initially examined by the naked eye, and hematoxylin and eosin (HE) staining was performed. Whole-slide images of lung lobes were captured using an Aperio CS2 slide scanner system (Leica Biosystems, Wetzlar, Germany) at a 20× magnification. The metastatic nodules of each slide were counted. All experiments were conducted according to the protocols approved by the Institutional Animal Care and Use Committee.

### Statistical analysis

The results are presented as the mean ± s.d. Statistical analyses were performed via GraphPad Prism5 and SPSS 16.0. Two-tailed Student’s *t* tests were used to assess the statistical significance of differences between the groups. The association between TM4SF1 expression and clinicopathological features was analyzed by the *χ2* test. Survival was estimated by the Kaplan–Meier method and the differences in survival between two groups were analyzed using the log-rank test. Multivariate analysis was performed using Cox’s proportional hazards model. Statistical significance was defined as *P* < 0.05 (not statistically significant (*n.s*.), *P* > 0.05; *P < 0.05; **P < 0.01; ***P < 0.001; ****,*P* < 0.0001).

## Supplementary information

Supplementary Table 1

Supplementary Table 2

Uncropped Western Blots

Checklist file for this paper

## Data Availability

All data needed to evaluate the conclusions are present in the paper. The uncropped western blots are shown in Supplementary Information as an ‘Uncropped Western Blots’ file.
