# Supplementary material for: TM4SF1 promotes esophageal squamous cell carcinoma metastasis by interacting with integrin α6
Source: Cell Death Dis. 2022 Jul 14;13(7):609. doi: 10.1038/s41419-022-05067-2 (PMC9283456; doi:10.1038/s41419-022-05067-2)
Supplement: Supplementary file 1 — Supplementary Table 1 [file 41419_2022_5067_MOESM1_ESM.docx]

**Supplementary Table 1.** TM4SF1 expression and clinicopathological parameters in 109 ESCC specimens.

| Characteristics | | *n* | TM4SF1 expression | | *χ^2^* | *P* |
| --- | --- | --- | --- | --- | --- | --- |
|  |  |  | High (%) | Low (%) |  |  |
| Specimen | |  |  |  |  |  |
| Adjacent normal tissue | | 109 | 38 (34.9%) | 71 (65.1%) | 17.639 | < 0.001^*^ |
| ESCC | | 109 | 69 (63.3%) | 40 (36.7%) |  |  |
| Gender | |  |  |  |  |  |
| Female | | 16 | 12 (75%) | 4 (25%) | 1.105 | 0.293 |
| Male | | 93 | 57 (61.3%) | 36 (38.7%) |  |  |
| Age | |  |  |  |  |  |
| > 60 | | 83 | 54 (65.1%) | 29 (34.9%) | 0.463 | 0.496 |
| ≤ 60 | | 26 | 15 (57.7%) | 11 (42.3%) |  |  |
| Smoking history | |  |  |  |  |  |
| Smoking | | 80 | 52 (65.0%) | 28 (35.0%) | 0.373 | 0.541 |
| None smoking | | 29 | 17 (58.6%) | 12 (41.4%) |  |  |
| TNM stage | |  |  |  |  |  |
| Ⅰ-Ⅱ | | 81 | 46 (56.8%) | 35 (43.2%) | 5.757 | 0.016^*^ |
| Ⅲ | | 28 | 23 (82.1%) | 5 (17.9%) |  |  |
| T classification | |  |  |  |  |  |
| T1 + T2 | | 56 | 38 (67.9%) | 18 (32.1%) | 1.028 | 0.311 |
| T3 + T4 | | 53 | 31 (58.5%) | 22 (41.5%) |  |  |
| N classification | |  |  |  |  |  |
| NO | | 67 | 37 (55.2%) | 30 (44.8%) | 4.885 | 0.027^*^ |
| Yes | | 42 | 32 (76.2%) | 10 (23.8%) |  |  |
| Differentiation | |  |  |  |  |  |
| Well | | 21 | 9 (42.9%) | 12 (57.1%) | 4.681 | 0.031* |
| Moderately/poorly | | 88 | 60 (68.2%) | 28 (31.8%) |  |  |
| Tumor size | |  |  |  |  |  |
| < 4 | | 42 | 21 (50%) | 21 (50%) | 5.205 | 0.023^*^ |
| ≥ 4 | | 67 | 48 (71.6%) | 19 (28.4%) |  |  |

Statistical analyses were performed by the Pearson *χ2* test.
*, *P* < 0.05 was considered significant.
