# Supplementary material for: TM4SF1 promotes esophageal squamous cell carcinoma metastasis by interacting with integrin α6
Source: Cell Death Dis. 2022 Jul 14;13(7):609. doi: 10.1038/s41419-022-05067-2 (PMC9283456; doi:10.1038/s41419-022-05067-2)
Supplement: Supplementary file 2 — Supplementary Table 2 [file 41419_2022_5067_MOESM2_ESM.docx]

**Supplementary Table 2.** Contribution of various potential prognostic factors to survival by Cox regression analysis in 109 ESCC specimens.

|  | Relative ratio | 95% Confidence interval | *P* |
| --- | --- | --- | --- |
| Age (years) | 1.342 | 0.679-2.653 | 0.398 |
| Gender | 1.166 | 0.549-2.476 | 0.69 |
| Tumor size | 1.111 | 0.645-1.913 | 0.704 |
| TMN stage | 2.511 | 1.365-4.619 | 0.003^*^ |
| Smoking history | 0.672 | 0.374-1.206 | 0.183 |
| T classification | 0.962 | 0.558-1.66 | 0.89 |
| N classification | 0.840 | 0.483-1.461 | 0.538 |
| Differentiation | 1.199 | 0.548-2.621 | 0.65 |
| TM4SF1 | 2.272 | 1.259-4.099 | 0.006^*^ |

Statistical analyses were performed by the log-rank test.
*, *P* < 0.05 was considered significant.
