# Supplementary figures and images for: TM4SF1 promotes esophageal squamous cell carcinoma metastasis by interacting with integrin α6
Source: Cell Death Dis. 2022 Jul 14;13(7):609. doi: 10.1038/s41419-022-05067-2 (PMC9283456; doi:10.1038/s41419-022-05067-2)

Figure 1C

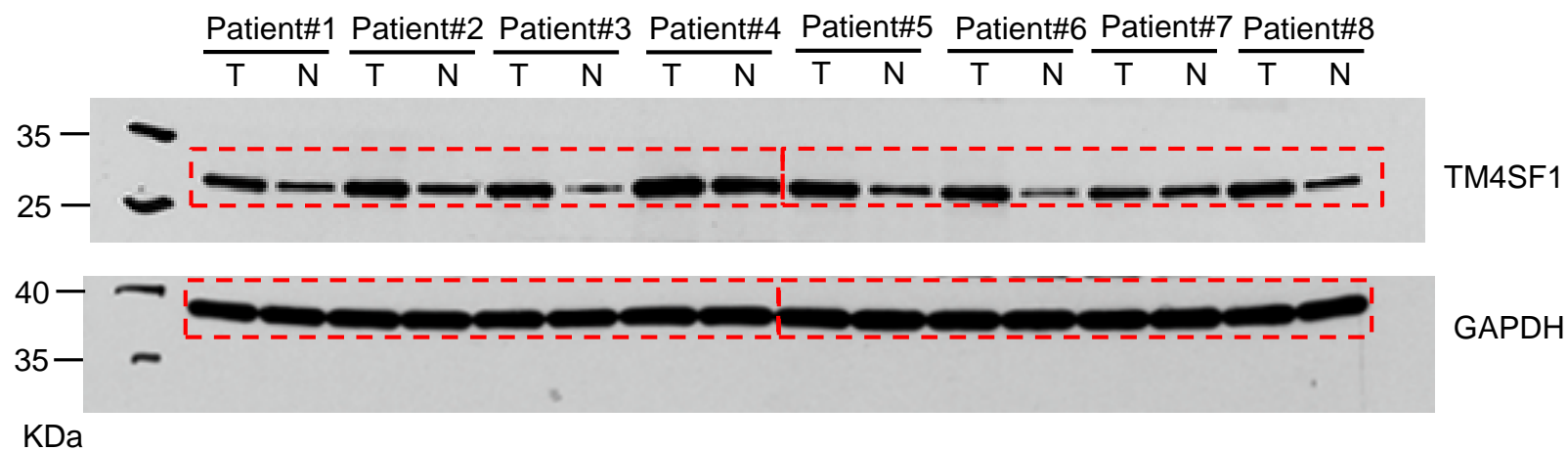

Figure 2A

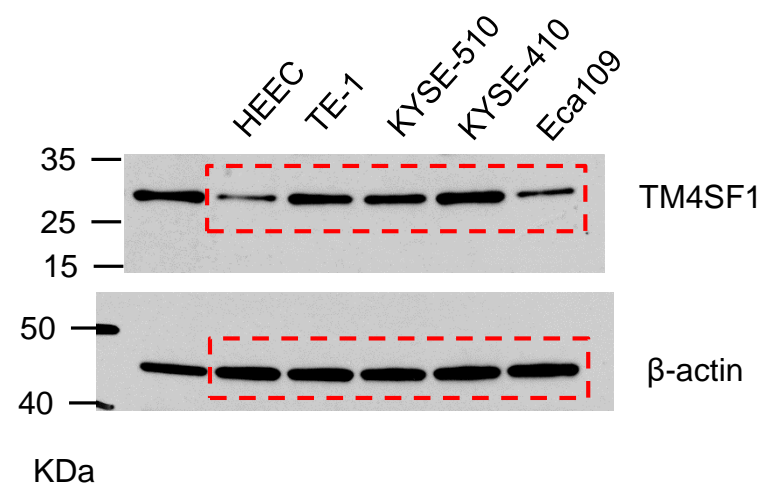

Figure 2B

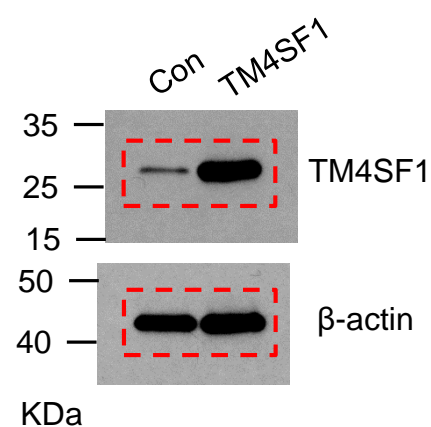

Figure 2D

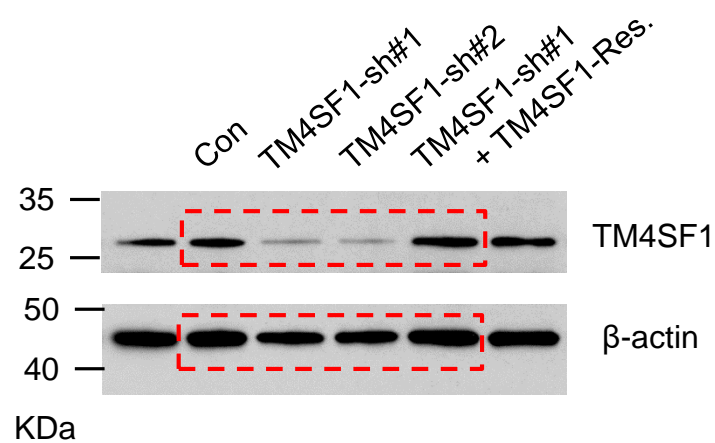

Figure 4A

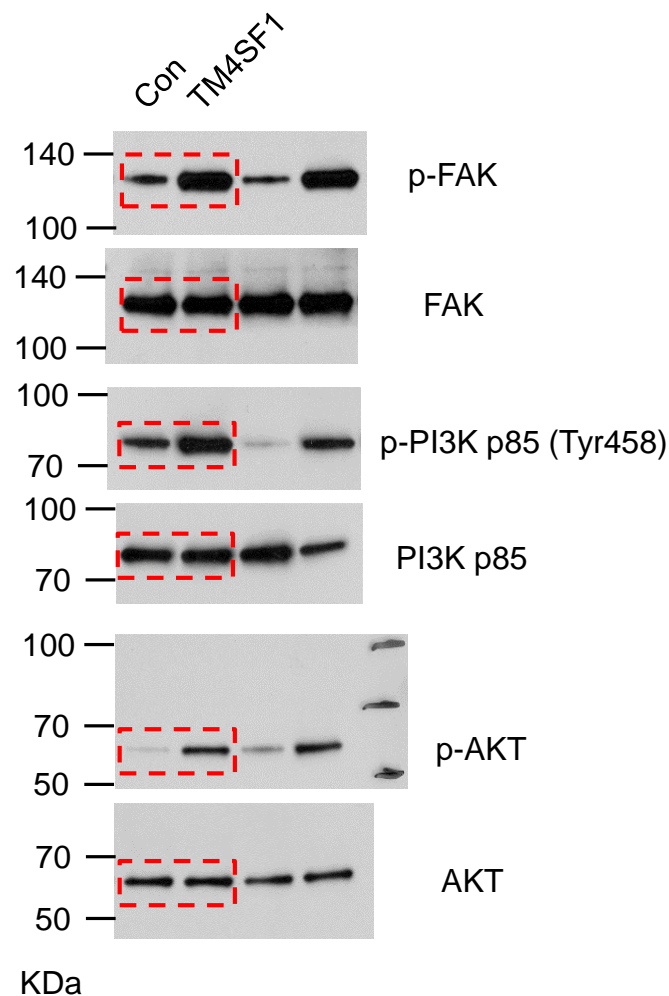

Figure 4C

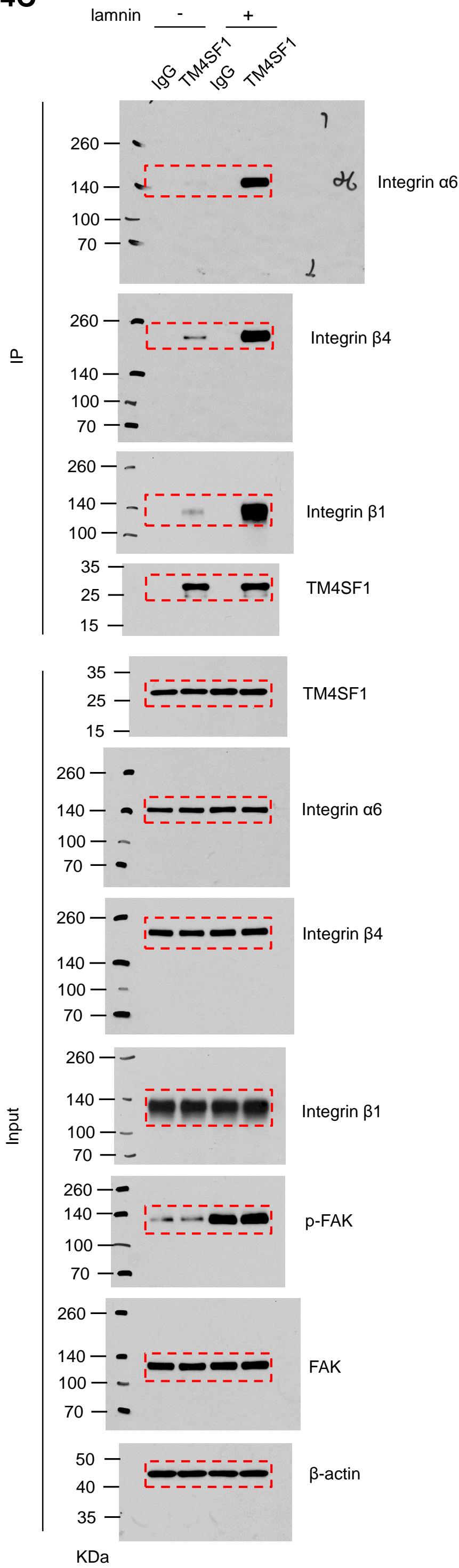

Figure 4D

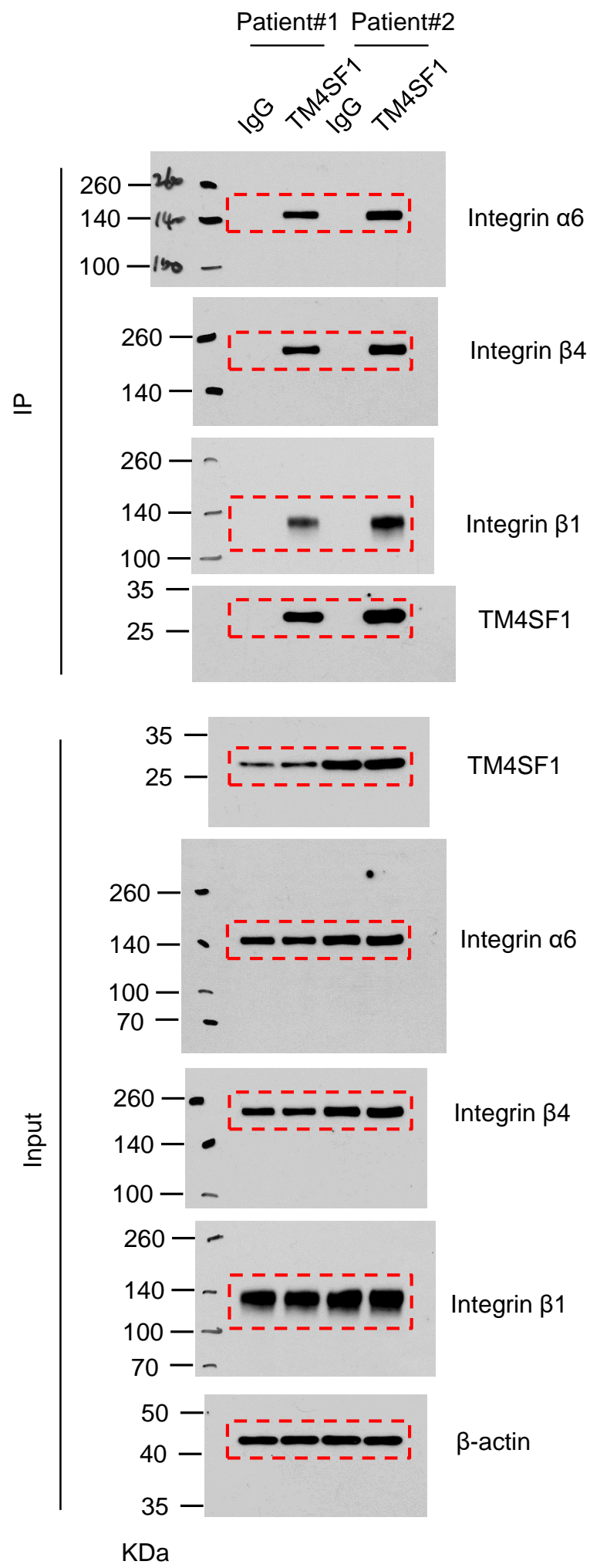

Figure 5A

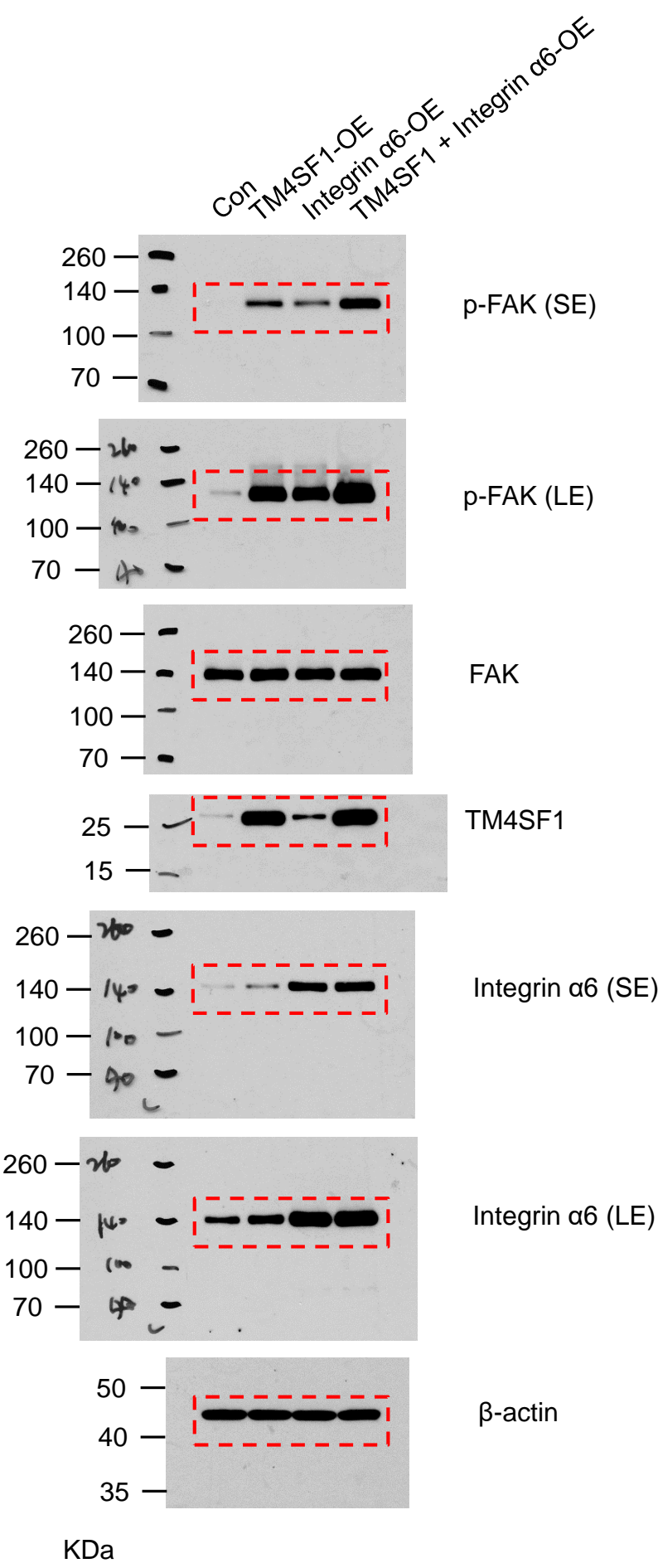

Figure 5B

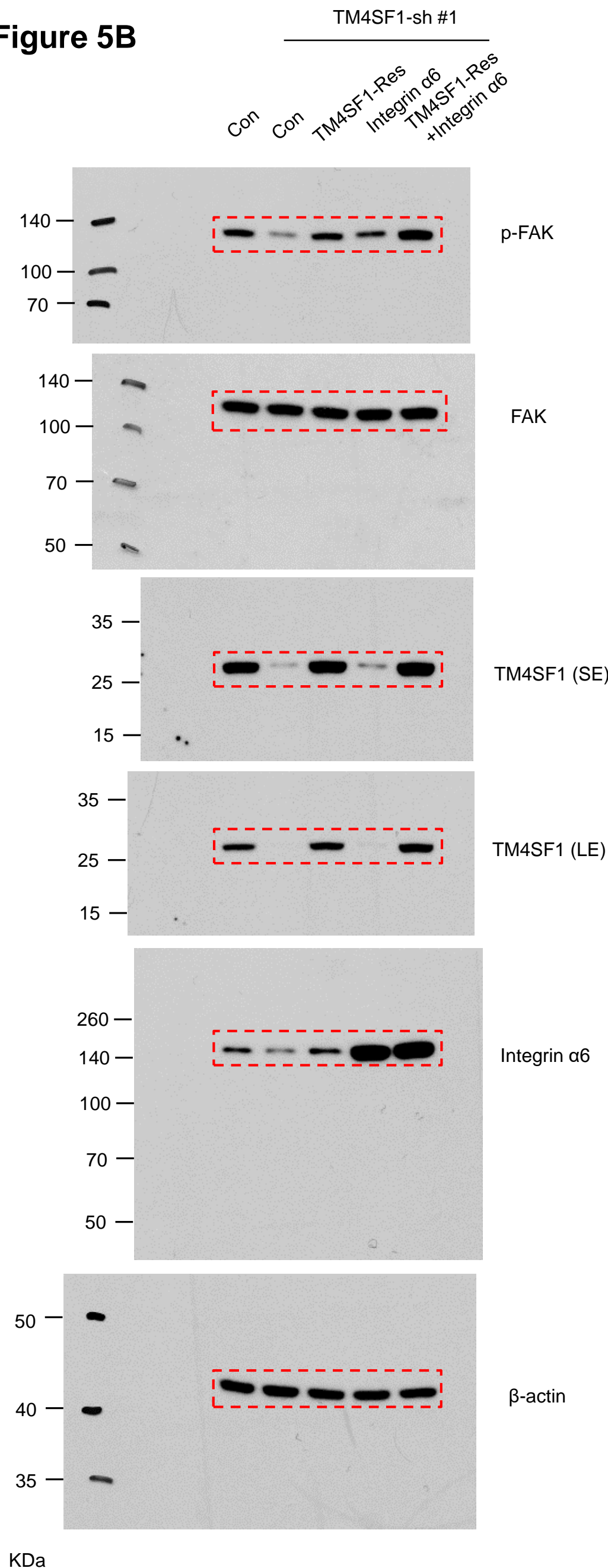

Figure 5C

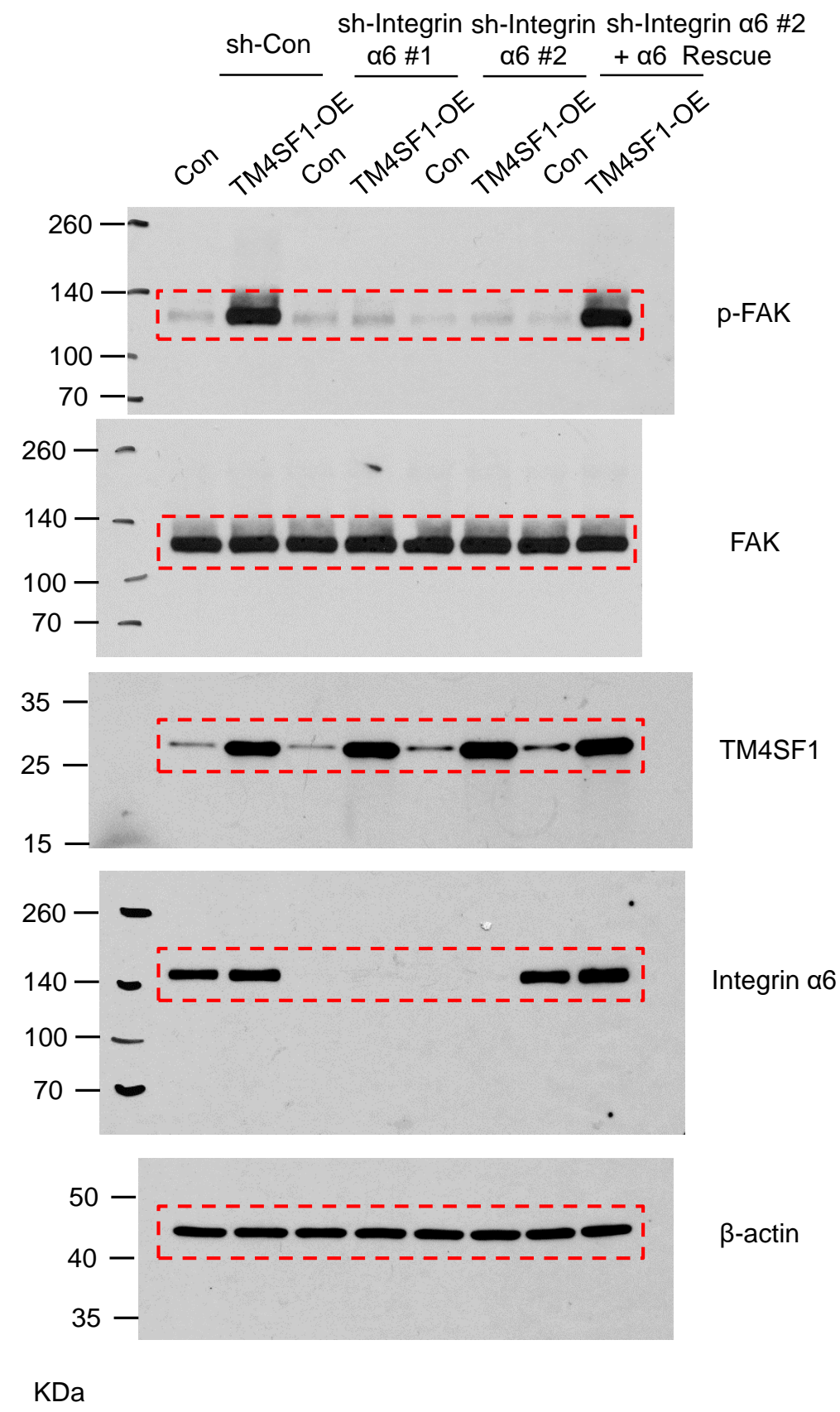

Figure 5D

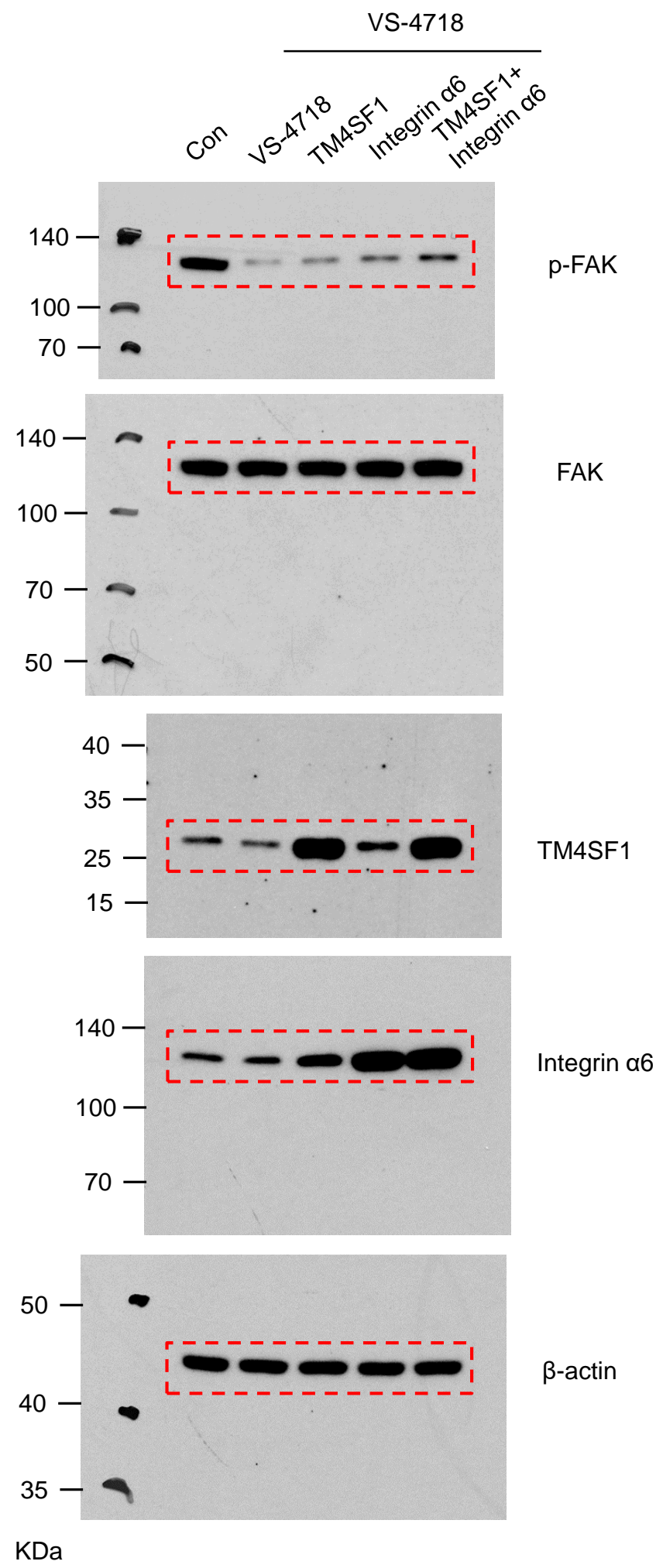

Supplement: Supplementary file 3 — Uncropped Western Blots [file 41419_2022_5067_MOESM3_ESM.pdf]
